# Developing Nanostructured Ti Alloys for Innovative Implantable Medical Devices

**DOI:** 10.3390/ma13040967

**Published:** 2020-02-21

**Authors:** Ruslan Z. Valiev, Egor A. Prokofiev, Nikita A. Kazarinov, Georgy I. Raab, Timur B. Minasov, Josef Stráský

**Affiliations:** 1 Institute of Physics of Advanced Materials, Ufa State Aviation Technical University, 12 K. Marx street, 450008 Ufa, Russia; giraab@mail.ru; 2 Laboratory of Mechanics of Advanced Bulk Nanomaterials, Saint Petersburg State University, Universitetskiy prospekt 28, Peterhof, 198504 St. Petersburg, Russia; egpro@mail.ru (E.A.P.); n.kazarinov@spbu.ru (N.A.K.); 3 Department of Traumatology and Orthopedics, Bashkir State Medical University, 3 Lenin street, 450008 Ufa, Russia; m004@yandex.ru; 4 Department of Physics of Materials, Charles University, Ke Karlovu 3, 121 16 Prague, Czech Republic; josef.strasky@gmail.com

**Keywords:** nanostructured Ti alloys, severe plastic deformation, enhanced strength and fatigue life, medical implants with improved design, shape-memory NiTi alloy, functionality

## Abstract

Recent years have witnessed much progress in medical device manufacturing and the needs of the medical industry urges modern nanomaterials science to develop novel approaches for improving the properties of existing biomaterials. One of the ways to enhance the material properties is their nanostructuring by using severe plastic deformation (SPD) techniques. For medical devices, such properties include increased strength and fatigue life, and this determines nanostructured Ti and Ti alloys to be an excellent choice for the engineering of implants with improved design for orthopedics and dentistry. Various reported studies conducted in this field enable the fabrication of medical devices with enhanced functionality. This paper reviews recent development in the field of nanostructured Ti-based materials and provides examples of the use of ultra-fine grained Ti alloys in medicine.

## 1. Introduction

Presently, Ti and its alloys represent the top choice when a combination of high strength, light weight, and affordable cost are required, such as in the area of medical device manufacturing. However, the clinical demands for implantable medical devices are growing rapidly, and nowadays new Ti alloys are being investigated in terms of their chemical composition optimization, manufacturing processes and modification of surface to meet the appropriate medical standards and comply with regulation [1,2]. One possibility to design and manufacture new materials with enhanced properties focuses on nanostructuring of metallic materials using the so-called severe plastic deformation (SPD) techniques, which have become a cutting edge and promising area in materials science and engineering [3,4].

Different SPD techniques are applied to refine grains in metallic materials to below micrometer range or even to the nanosized range. SPD techniques are also efficient for the formation of nanoclusters and nanoprecipitates of secondary phases, enhancing the mechanical and functional properties of the materials [4,5]. A whole variety of SPD techniques have been developed and put forward to provide very high strains (ε > 5) under high applied pressure, such as accumulative roll bonding (ARB), including multiple forging, twist extrusion, and others [6,7,8]. However, equal channel angular pressing (ECAP) and high pressure torsion (HPT), introduced already in the pioneering works [3], remain the most used methods for the production of ultrafine-grained (UFG) materials. Principles of these techniques, developed devices and microstructure evolution during processing steps have been thoroughly reviewed in numerous studies [3,4,5,6,7,9,10]. Recently, these deformation techniques have been further upgraded for practical application [11,12]. 

Nanostructuring of metallic materials increases material strength due to work hardening and grain refinement [13,14], consequently, fatigue life can be also significantly increased by microstructure refinement [15]. Understanding material processing by SPD techniques is essential for designing of medical devices with improved functionality as it not only improves mechanical properties but also affects corrosion and biomedical properties [16,17,18]. Improved strength and enhanced biomedical response of a nanostructured material can be efficiently used in dental implants; a stent of such permanent implant manufactured from nanostructured Ti can be significantly smaller due to the increased strength and therefore less harmful for a patient [19].

Recently, materials scientists have been exploring possibilities of improved interaction of nanostructured materials with body tissues, for instance bones. In this respect, surface modifications of bulk nanomaterials demonstrate encouraging results [17,18,20,21]. These improvements provide the possibility for development and design of implantable medical devices that perform better and provide improved functionality in comparison to their counterparts manufactured from common coarse-grained materials. This review article outlines the progress in engineering of advanced nanostructured Ti alloys and medical implants/devices manufactured from those advanced materials.

## 2. SPD Processing of Nanostructured Titanium Materials

### 2.1. Commercially Pure Ti

The first studies devoted to Ti-based materials potentially applicable in medicine were applied to commercial purity titanium (CP Ti) due to its high biocompatibility with living tissues [22]. Unparalleled biocompatibility of Ti was the main interest of many clinical studies of medical devices and tools applied in traumatology, orthopedics, and dentistry. Unfortunately, CP Ti is characterized by reduced strength when compared to other metallic materials used in biomedical devices such as steels or cobalt-based alloys. Achieving higher strength level is possible by alloying or thermo-mechanical processing, but then the Ti-based materials usually lose their biometric response or fatigue performance. Therefore, SPD processing was considered as an alternative strategy proving that nanostructuring of CP Ti may become a novel approach to improve the mechanical properties of this material to achieve its high-performance [13,17,19,20,23]. Apart from enhancing mechanical properties, this strategy is also advantageous in improving the biological response of the surface of the CP titanium based products [18,20].

The first results on nanostructured CP Ti Grade 4 (O–0.34%, Fe–0.3%, C–0.052%, N–0.015%, all in wt.%, balance–Ti] were achieved by Valiev et al. aiming on manufacturing rods with significantly enhanced mechanical properties and superior biomedical response for the fabrication of dental implants [19]. The processing route involved equal-channel angular pressing (ECAP) as an SPD technique [9] followed by thermo-mechanical treatment by forging and, finally, drawing. Continuous SPD processing by ECAP-Conform (ECAP-C) and subsequent drawing, was capable of producing rods with the diameter of 7 mm and the length of 3 m with homogeneous ultrafine-grained (UFG) structure along the entire length of the rods [23,24]. Furthermore, ECAP-Conform represents an economical SPD-based fabrication procedure for mass production of ‘nanoTi’.

**Figure 1:**
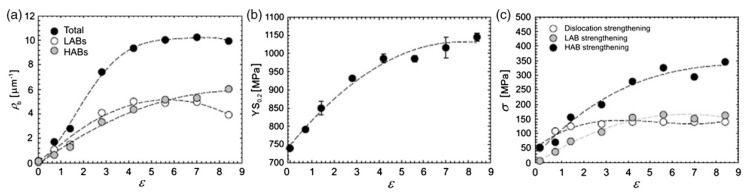
Influence of ECAP-C strain on (**a**) grain boundary (GB) density, (**b**) yield strength and (**c**) the contribution of various strengthening mechanisms [21].

After combined severe plastic deformation and thermo-mechanical processing, the grain size was significantly reduced from 25 µm in the initial Ti rods to 150 nm in the processed material. Figure 1 illustrates the effect of ECAP-C strain on the density of high-angle boundaries (HAB) and mechanical strength of CP Ti Grade 4 [21].

Table 1 shows the improved mechanical properties of CP Ti after nanostructuring by ECAP and subsequent thermomechanical treatment. The strength of the nanostructured titanium is doubled when compared to the conventional CP titanium. The increase in strength was achieved without reduction of ductility (total elongation to failure is above the limit of 10%), which is otherwise commonly observed after intensive drawing or rolling.

Fatigue tests of conventional and nanostructured CP Ti were conducted in air at room temperature in accordance with ASTM E 466-96 with the loading frequency of 20 Hz and R = 0.1. Table 1 shows that the fatigue strength of nanoTi [17,24] after one million cycles is almost doubled when compared to the conventional CP titanium and even exceeds the fatigue performance of the Ti-6Al-4V alloy [22,25]. Significant enhancement of fatigue properties and improved strength of nanostructured Ti allow us to produce smaller sizes of implants and therefore to reduce the extent of a surgical intervention (see also Section 3).

CP Ti is known for its considerable biocompatibility which results from the presence of the protective oxide film. Titanium dioxide TiO_2_ forms naturally on the surface of CP Ti and represents a stable protective layer on that a mineralized bone matrix can be attached. This film is usually 5–10nm thick and biologically inert, thus it prevents a potentially negative reaction between the surrounding body environment and the metal [22]. 

NanoTi with UFG structure containing high density of non-equilibrium grain boundaries achieved by SPD is also characterized by significantly increased internal energy of the material [3]. This fact may result in considerable change in the morphology of the oxide film on the material surface. NanoTi with polished surface exhibits improved biological reaction of the surface as confirmed by recent studies in a series of experiments through cytocompatibility tests using mouse fibroblast cells [20,26,27,28,29]. At the same time, additional improvement of biomedical properties of nanostructured titanium can be achieved by dedicated surface modifications such as chemical etching or bioactive coatings [17,18].

### 2.2. Titanium Alloys

Two-phase (α + β) titanium alloys such as Ti-6Al-4V and Ti-6Al-7Nb continue to be the most important metallic materials in the dental and orthopedic fields due to their excellent mechanical properties and satisfactory biocompatibility [2,22,30,31].

Several recent studies reported improved mechanical and functional properties of nanostructured titanium alloys.

Microstructure and mechanical properties of Ti-6Al-4V ELI (extra low interstitial alloys for medical applications) prepared by SPD are reported in [15,32,33]. Round rods of the two-phase alloy with the diameter of 40 mm (Intrinsic Devices Company, San Francisco, CA, USA) and with chemical composition: Ti–base, Al–6.0%; V–4.2%; Fe–0.2%; O–0.11%; N–0.0025%; H–0.002%, C–0.001% (wt.%) had the grain size of about 8 µm in a cross-section and 20 µm in a longitudinal section. X-ray diffraction analysis proved that the volume fractions of α and β phases were approximately 85% and 15%, respectively. 250 mm length rods were processed in two steps. The rods were subjected to ECAP via route Bc at 600 °C and subsequently extruded, altogether with total strain of 4.2 [33]. The extrusion steps were carried out at 300 °C with the last pass at room temperature for additional strengthening. The rods with the diameter of 18 mm and length up to 300 mm were produced. The rods were finally annealed in the temperature range from 200 °C to 800 °C for 1 h and subsequently cooled in air.

Transmission electron microscopy (TEM) studies showed that SPD leads to a complex UFG structure containing refined grains and subgrains with a mean size of about 300 nm.

**Figure 2:**
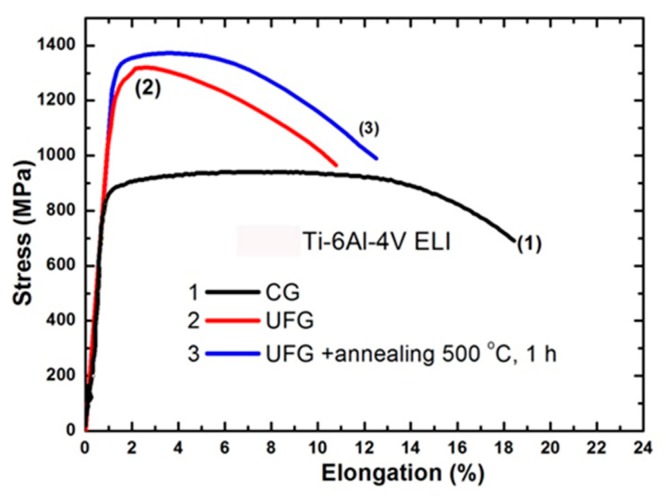
Engineering stress−strain tensile curves of the Ti-6Al-4V ELI alloy: coarse-grained material (initial) (1); UFG condition (2) and UFG condition after annealing at 500 °C (3).

Stress–strain curves for the initial coarse-grained and UFG material shown in Figure 2 demonstrate that the alloy after grain refinement by SPD underwent significant strengthening. Tensile elongation of the UFG material (curve 2) is reduced from 17% to 9%. Strength/ductility trade off, however, improved after subsequent annealing at 500 °C. The results of tensile tests correspond to the measurement of microhardness [32,33].

In accordance with [10], enhancement of the ductility in the UFG material by annealing is clearly associated with a decrease of internal elastic stress and dislocation density. Simultaneous additional strengthening of the alloy can be explained by the observed decrease in content of metastable β-phase after cooling from the annealing temperature. Its volume fraction in the UFG alloy annealed at 500 °C can be higher than before annealing, as shown in [10], due to quenching from the annealing temperature. Despite no visible particles of any secondary phase, aging processes might have caused grain boundary segregations associated with additional improvement of the properties of the annealed UFG material [34]. 

Fine tuning of mechanical properties by annealing after the SPD processing is limited mainly by grain growth occurring at elevated temperatures. Thermal stability of UFG structure of commercially pure Ti follows classical grain growth depending on temperature via Arrhenius equation [35] and limited to approximately 450 °C [36]. Nanostructured α + β exhibit enhanced thermal stability up to 550 °C [37].

**Figure 3:**
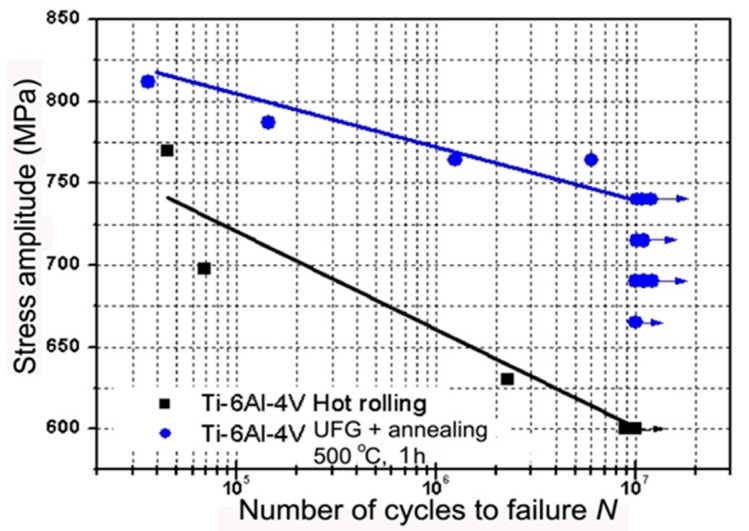
Fatigue test results of initial coarse-grained material and UFG material after annealing at 500 °C, 1 h.

Fatigue properties of the Ti-6Al-4V ELI alloy with UFG structure were investigated. High strength and enhanced ductility (1370 MPa and 12%) after SPD processing and subsequent annealing at 500 °C; resulted in an enhancement of fatigue limit to 740 MPa after 10^7^ cycles in comparison to 600 MPa in the initial coarse-grained condition (Figure 3) [32]. 

The fatigue limit of the Ti-6Al-4V alloy in UFG condition reported in [32] tested by rotating bending was slightly higher than the values in [32,38] proving that measured fatigue properties depend on the choice of the measurement technique.

Achieved results show that high strength can be achieved in UFG Ti-6Al-4V ELI alloy by processing by ECAP and subsequent thermo-mechanical treatment. Selection of SPD regimes and adjustment of processing parameters of SPD processing such as temperature, strain rate and strain allow us to manipulate the grain boundary structure and phase morphology in the two-phase UFG alloy. As the result, the best combination of strength and ductility can be achieved along with the improved fatigue endurance limit. Enhancement of strength and ductility of the biomedical Ti-6Al-7Nb alloy was reported in another comprehensive study [39]. In comparison to Ti-6Al-4V, the Ti-6Al-7Nb alloy represents a better choice for biomedical use due to avoiding the toxic vanadium [40]. This study shows that processing by ECAP and consequent thermo-mechanical treatment causing formation of UFG structure results in high strength (1400 MPa) and ductility (elongation of 10%). These achieved properties are attractive for designing, developing and manufacturing of high-performance medical devices and implants.

Considering that vanadium and partly also aluminum are rather toxic elements and, simultaneously, that reducing of the Young’s modulus is required for avoiding so-called stress-shielding [39], the development of brand new biomedical alloys represents a current relevant challenge for researchers. A new generation of titanium alloys must provide improved strength, better biocompatibility, and lower Young’s modulus than Ti6Al4V alloy. Current research focuses on new alloying systems, in particular Ti-Nb and Ti-Mo. 

Given the above mentioned requirements, the interest is drawn to titanium alloys containing high content of the β phase, because this phase is characterized by lower Young’s modulus in the range of 55–90 GPa, and thus exhibit lower stress shielding [39,41,42,43]. Moreover, these Ti alloys are designed to contain only non-toxic constituents such as Nb, Mo, Zr, and Ta. On the other hand, these materials are characterized by comparatively low strength, because the lowest Young’s modulus is obtained only in solution treated single phase β-Ti alloys. Achieving low Young’s modulus and high strength simultaneously is a challenging task. Ageing treatments that induce a fine and uniform precipitation of ω and α phase components provides significant strengthening. On the other hand, this inevitably increases the Young’s modulus of the alloy [41,42,43]. Only few studies present successful results in development of thermal treatments without detrimental effect on some of the relevant mechanical properties [44,45].

**Figure 4:**
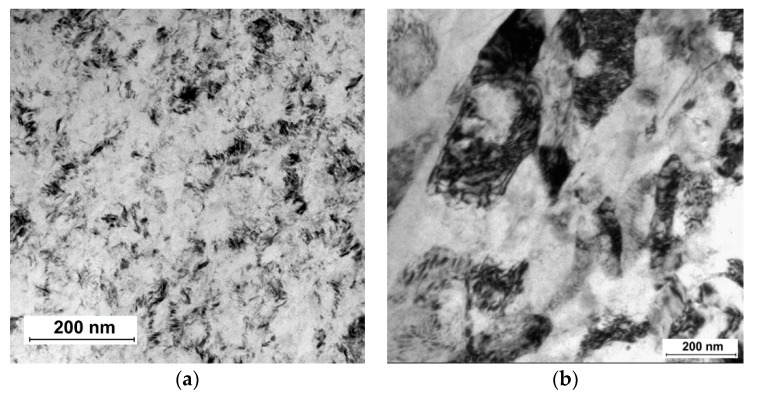
Microstructure of (**a**) Ti15Mo alloy prepared by HPT and (**b**) Ti-35Nb-6Ta-7ZZr alloy prepared by ECAP (cross-section).

Advancements in the areas of orthopedics and dentistry called for new strategies for development of new generation of β-Ti alloys with reduced Young’s modulus and high strength, which would be more suitable for such applications. Recently, SPD processing has been proposed to fabricate nanocrystalline β-Ti alloys with high strength, low modulus of elasticity and excellent biocompatibility [46,47,48,49,50,51]. Nanostructuring of these alloys leads to improved strength due to grain refinement and substructure evolution [52]. In particular, solution treated β-Ti Ti15Mo alloy, which is qualified for medical use, can be significantly refined by HPT as demonstrated in Figure 4a. Grain size can be decreased well below 100 nm [53]. Significant disadvantage, apart from limited size of HPT samples, is formation of deformation induced ω phase causing sharp increase of elastic modulus [54]. Subsequent aging of UFG Ti15Mo alloy leads to two-phase α + β structure which is also characterized by increased modulus of elasticity [55,56,57]. More promising is using Ti-Nb-Ta-Zr based alloys which are less prone to ω phase formation. Ti-29Nb-13Ta-5Zr alloy prepared by HPT exhibited increased yield stress from 550 to 800 MPa with unchanged elastic modulus [58,59]. Significant microstructure refinement was recently also achieved in Ti-35Nb-6Ta-7Zr biomedical alloy by ECAP (Figure 4b).

Microstructural refinement in β-Ti alloys can be also enhanced by multiple twinning and/or martensitic transformation β → α’’ [60]. The nanocrystalline β-Ti alloys also display excellent in vitro biocompatibility as shown by enhanced cell attachment and proliferation [48]. These novel nanocrystalline β-Ti alloys have high chances to meet the challenge of next-generation implant material with significant prospects in load bearing biomedical applications.

### 2.3. Nanostructured NiTi Shape Memory Alloys

NiTi alloys exhibit unique mechanical behavior—shape memory effect (SME) and superelasticity, which arise from a transformation between martensite and austenite phases [61,62]. NiTi alloys are important materials which are already used in advanced medical devices due to the above mentioned mechanical properties and, additionally, due to functional properties such as good biocompatibility and corrosion resistance in vivo [61,63]. At the same time, new, advanced applications will require enhanced properties (higher strength, higher recovery strain and stress, etc.) of NiTi shape memory alloys. 

During the past two decades, there has been interest in the application of SPD methods to NiTi alloys because the formation of nanocrystalline and UFG structures allows enhancing mechanical and functional properties in comparison to coarse grained materials [63,64]. 

**Figure 5:**
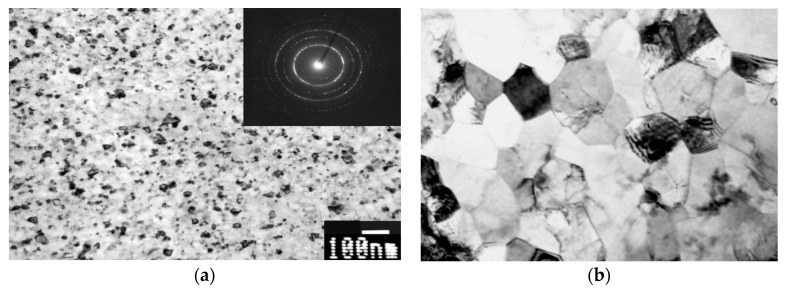
Microstructure of (**a**) NC and (**b**) UFG NiTi alloys processed by HPT and ECAP, respectively.

HPT processing of NiTi alloys leads to a transformation from crystalline to amorphous phase. Microstructural changes in deformed NiTi during thermal treatment are of key interest as they are responsible for the shape memory effect [65,66]. During following thermal treatments nanocrystalline (NC) structure can be obtained in NiTi alloys via crystallization process (Figure 5) [64,67]. Nanocrystalline NiTi alloys with grain size about 20 nm demonstrate very high strength up to 2000 MPa [64]. 

Equal channel angular pressing is another SPD processing technique applied for producing uniform UFG structure in bulk NiTi alloys. The ECAP processing of NiTi at 400–450 °C results in formation of UFG structure with grain size of about 200 nm (Figure 5). 

**Figure 6:**
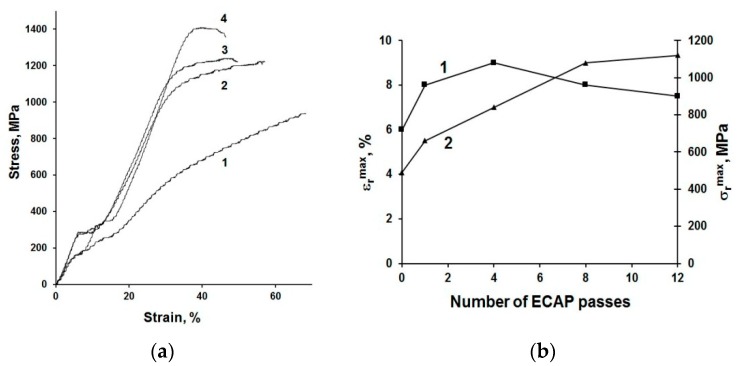
Mechanical properties of NiTi alloy in CG condition and after ECAP. (**a**) Engineering stress–strain curves for tensile tests in CG state (1) and after ECAP using 4 (2), 8 (3) and 12 (4) passes and (**b**) functional properties (ε_r_^max^ and σ_r_^max^) as a function of number of ECAP passes [61].

UFG structure formation leads to significant improvement of mechanical and functional properties of NiTi-based alloys [64,68,69,70]. The ultimate tensile strength (UTS) of UFG NiTi alloy attains 1400 MPa, which is 50% higher than in CG alloys; and the yield stress (YS) increases after ECAP from 500 MPa to 1100 MPa (Figure 6a). The functional shape-memory effect of NiTi after ECAP is also improved (Figure 6b). The maximum completely recoverable strain ε_r_^max^ increases from 6% (in CG state) to 9% after ECAP and the maximum recovery stress σ_r_^max^ reaches 1120 MPa, which is twice more than the level of CG alloys (about 500 MPa) [69]. UFG structure formation in Ni-rich NiTi alloys by ECAP results in an emergence of superelasticity at temperature close to the human body temperature. Superelasticity in UFG NiTi is characterized by a narrow mechanical hysteresis and low residual strain [71].

The high-strength NC and UFG NiTi alloys with improved functional characteristics are very promising for medical applications in particular for manufacturing of stents, embolic protection filters, guide wires, and other peripheral vascular devices (see Section 4). 

## 3. Design of Miniaturized Implants

Enhanced mechanical properties of nanostructured metals allow development of medical implants with better design, for instance with a more subtle design which is less harmful for human body [17].

Application of stronger nanostructured CP Ti instead of common CG Ti, allows for altering the design of devices. Recently, detailed computations were conducted to analyze the possible geometries of miniplates for maxillofacial surgery manufactured from nanostructured Ti [72].

CP Ti miniplate specified by ASTM F 67, was considered by Conmet Company (Moscow, Russia) as the benchmark for redesigning the product dimensions of mini-plates manufactured from nanostructured CP Ti. The mechanical properties in a cross-section of a newly designed plate were calculated with the use of estimates of the fatigue performance limit for coarse-grained Grade 4 CP Ti and nanostructured Grade 4 CP Ti. In practical use, the mini-plates are subjected to bending loads, therefore bending strength of mini-plates from conventional and nanostructured CP Ti was compared. The result indicates that the plate from nanostructured Ti has significantly improved bending strength and therefore, it is clearly advantageous over the standard device currently manufactured from CG Ti.

Recently, three-dimensional finite element models (FEM) were developed using CAE software (KOMPAS-3D v15, ASCON Group, Saint Petersburg, Russia) and then imported into ANSYS Workbench 18.2 (ANSYS Inc., Canonsburg, PA, USA) [73] for geometry analysis of nanoTi dental implants. In addition to static strength, calculations of virtual fatigue testing were carried out using the built-in fatigue module. For all tested models, mesh sensitivity testing was performed in order to obtain mesh-independent results. 

The following procedure was used to assess possible ways to miniaturize the implants. The device with a standard geometry was assumed to be made from the conventional coarse-grained CP Ti. The model was designed in a way to obtain nearly critical stress state both in terms of static and fatigue failure. Afterwards, the same loading was applied to a model with reduced dimensions but with the properties of nano CP Ti.

**Figure 7:**
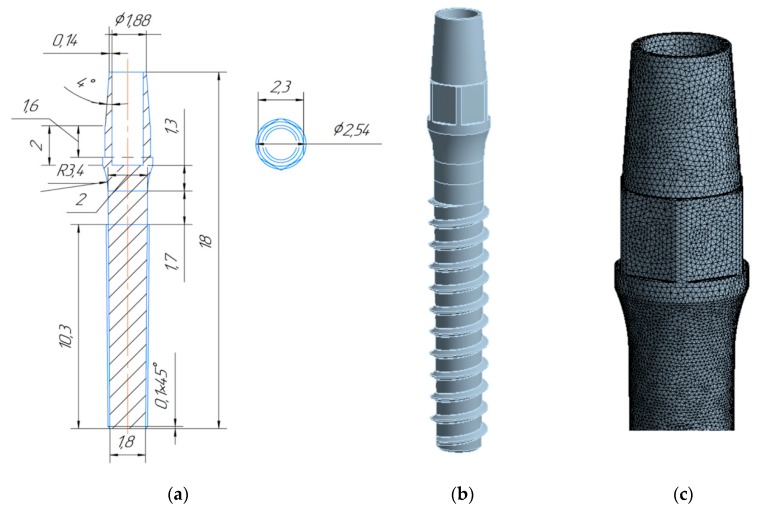
Geometry of the dental nanoimplant: (**a**) technical drawing with dimensions in mm; (**b**) 3D model; (**c**) enlarged FEM mesh.

A one-stage dental implant with generic geometry was considered in the study. The shape of the implant is similar to the implant geometry produced from nano CP Ti by company Timplant s.r.o. (Ostrava, Czech Republic) [74]. Figure 7 shows a technical drawing of the geometry of this nanoimplant with a corresponding numerical model.

**Figure 8:**
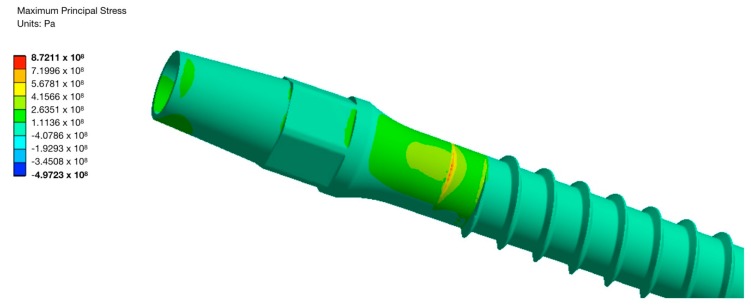
Maximal principal stress for the UFG Ti implant with a 10% reduced diameter and 67.75 N force.

The applied loading scheme was inspired by the testing procedures used in the ISO 14801 standard. The performed calculations revealed that application of the nanoTi allows reduction of the diameter of implant by at least 10%, while 20% diameter reduction leads to an unacceptable decrease of the device’s fatigue strength. Maximal principal stress zone for the implant with the diameter reduced by 10% loaded with a 67.75 N force is shown in Figure 8.

## 4. Fabrication and Tests of Medical Nanoimplants

**Figure 9:**
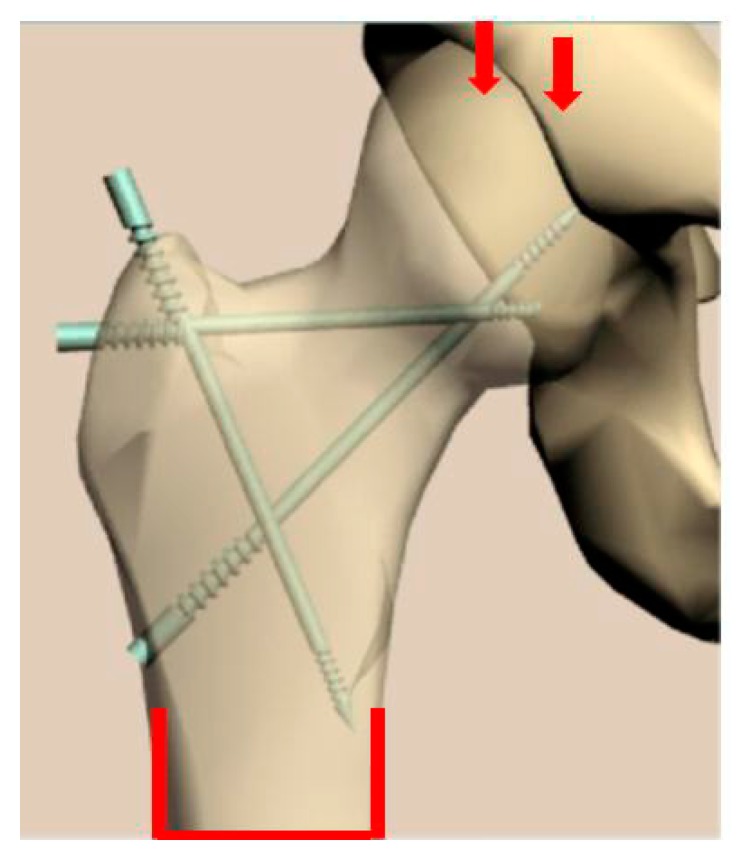
The image of the hip after the insertion of reinforcing implants.

**Figure 10:**
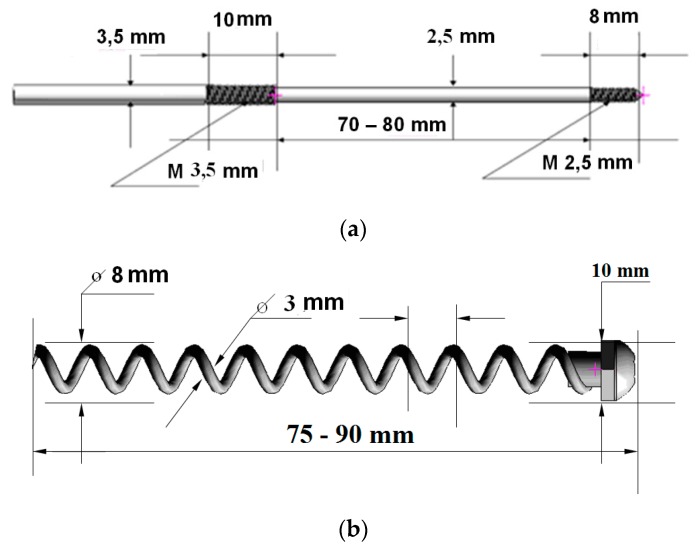
Two types of the implant systems used: ((**a**) a pin; (**b**) a spiral) and their application using the INSTRON 5982 dynamometer.

**Figure 11:**
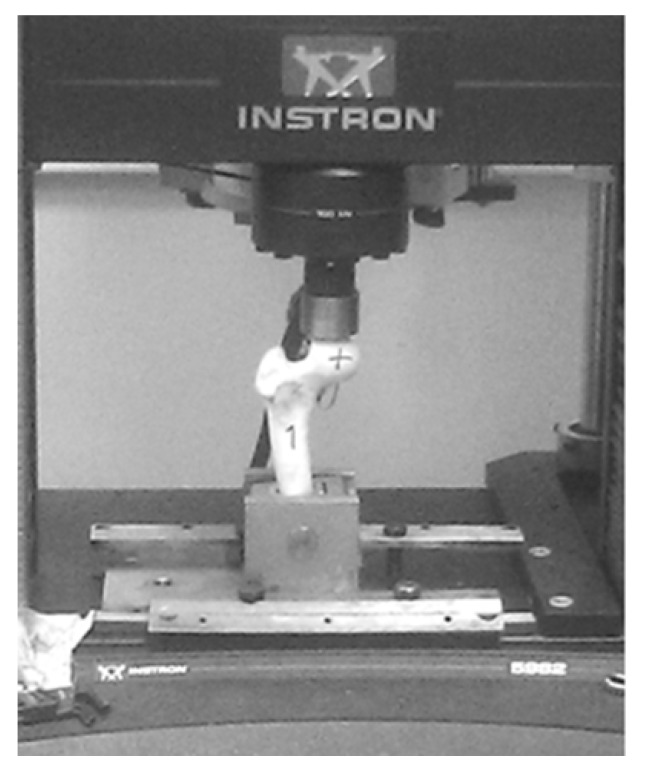
Testing procedure of the reinforced hip sample.

Recently, manufacturing and successful testing of several medical implants fabricated from nanostructured Ti have been considered in detail [17]. Another example of the innovative development is the manufacturing and testing of the implant pins designed for surgery in the bone tissue of the hip, which increases bone strength and prevents its fracture (Figure 9) [75]. The pins of two types (Figure 10) were produced from nanostructured Ti rods of 3 mm diameter with very high strength (σ = 1300 MPa). These implants were used to study their effect on the bone strength of the hip, which was evaluated by means of bench testing [76]. For this purpose, a special device (Figure 11) was used to analyze the mechanical properties of implant systems under compression along the axis of the hip. Such systems were subjected to a defined load along the axis of the hip, as well as in the perpendicular direction with a force directed to the region of the greater trochanter to complete fracture at a rate of 5 mm/min using the INSTRON 5982 (Instron®, High Wycombe, Buckinghamshire, UK) multipurpose one pin dynamometer. A total of 3 systems were studied: three pins, a spiral, and a spiral + pin system. As a result [76], the use of different implants demonstrated high efficiency in improving the strength of bone tissue in the hip. In particular, the use of a spiral and a pin in the bone-implant system made it possible to increase the axial load resistance by 72.6% in comparison to the tests excluding implants. This demonstrated the prospect of integration of surgical reinforcement of the hip made of nano CP Ti into clinical practice to prevent broken bones. 

Another interesting example of the innovative application is the removable clipping device for blood vessels, tubular structures, and soft tissues fabricated from UFG NiTi with enhanced shape-memory effect and designed for bleeding control during laparoscopic operations. This device has been created and tested in collaboration between Ufa State Aviation Technical University (USATU) and National University of Science and Technology “MISIS” [24].

The conducted tests demonstrated that the removable clipping devices produced from UFG NiTi alloy obtain several advantages when compared to the standard counterpart. Table 2 provides the most important properties of the clipping device for the UFG and conventional CG alloy. The maximum opening angle of the jaws, at which no residual deformation was observed, increases up to 160°, which is significantly higher than that of benchmark CG alloy. The value of the reversible shape memory effect (up to 4 mm) and the maximum rated force that develops at triggering the clipping device (up to 0.9 N) also doubles in the product from UFG alloys.

Higher completely recoverable strain allows for a more convenient shape of the clipping device to be used for laparoscopy and manipulation; it also helps to reduce the diameter of a laparoscope tube, i.e., to create more comfortable surgery conditions for a doctor and a patient. Due to higher deformation with a reversible shape memory effect, it is possible to improve non-invasiveness when removing the clipping device. Increased maximum recovery stress provides high force that develops at triggering the clipping device, high reliability of tissue crimping and fixation, and also makes it possible to reduce the weight of the clipping device.

## 5. Conclusions

Recent studies have proven that nanostructuring of titanium materials by means of severe plastic deformation (SPD) techniques achieving grain refinement, increase of dislocation density, dissolution, and formation of secondary phase precipitations allows for considerable improvement of the strength and fatigue properties. In the present paper the advantages of nanostructuring were demonstrated for CP Ti, Ti alloys including new β-Ti alloys as well as the NiTi alloy with shape memory effect. The approaches to computer design of a number of miniaturized medical implants made from high-strength nanomaterials have been suggested. In addition, the paper includes the examples of manufacturing and tests of selected advanced medical devices for traumatology and surgery from Ti nanobiomaterials. Taking into account the results of recent studies on surface modification, including chemical etching of nanometals and deposition of bioactive coatings, it is assumed that the developments of Ti-based nanomaterials opens new possibilities for advanced medical implants and devices with improved design and functionality.

## Figures and Tables

**Table 1:** Mechanical properties of coarse-grained (CG) and nanostructured CP Grade 4 Ti. Annealed Ti-6Al-4V ELI (extra low interstitials) alloy for comparison.

State	Processing	UTS, MPa	YS, MPa	Elongation, %	Reduction Area, %	Fatigue Strength at 10^6^ Cycles
1	Initial CG Ti	700	530	25	52	340
2	nanoTi	1240	1200	12	42	620
3	Annealed Ti-6Al-4V ELI	940	840	16	45	530

**Table 2:** Service characteristics of the clipping device produced from the NITi alloys.

Material	Opening Angle of the Jaws, °	Opening of the Jaws at Reversible Shape Memory Effect, mm	Max Rated Force of the Clipping Device, H
CG	<110	2	0.44
UFG	160	4	0.9
